# Disentangling the effects of plant species invasion and urban development on arthropod community composition

**DOI:** 10.1111/gcb.15091

**Published:** 2020-04-16

**Authors:** Wendy A. M. Jesse, Jasper Molleman, Oscar Franken, Mark Lammers, Matty P. Berg, Jocelyn E. Behm, Matthew R. Helmus, Jacintha Ellers

**Affiliations:** ^1^ Department of Ecological Science – Animal Ecology Vrije Universiteit Amsterdam Amsterdam The Netherlands; ^2^ Institute for Evolution and Biodiversity University of Münster Münster Germany; ^3^ Groningen Institute for Evolutionary Life Sciences – Community and Conservation Ecology University of Groningen Groningen The Netherlands; ^4^ Integrative Ecology Lab Center for Biodiversity Department of Biology Temple University Philadelphia PA USA

**Keywords:** Anthropocene, *Antigonon leptopus*, coralita, exotic species, feeding guilds, functional traits, land use change, multistressor effects

## Abstract

Urban development and species invasion are two major global threats to biodiversity. These threats often co‐occur, as developed areas are more prone to species invasion. However, few empirical studies have tested if both factors affect biodiversity in similar ways. Here we study the individual and combined effects of urban development and plant invasion on the composition of arthropod communities. We assessed 36 paired invaded and non‐invaded sample plots, invaded by the plant *Antigonon leptopus*, with half of these pairs located in natural and the other half in developed land‐use types on the Caribbean island of St. Eustatius. We used several taxonomic and functional variables to describe community composition and diversity. Our results show that both urban development and *A. leptopus* invasion affected community composition, albeit in different ways. Development significantly increased species richness and exponential Shannon diversity, while invasion had no effect on these variables. However, invasion significantly increased arthropod abundance and caused biotic homogenization. Specifically, uninvaded arthropod communities were distinctly different in species composition between developed and natural sites, while they became undistinguishable after *A. leptopus* invasion. Moreover, functional variables were significantly affected by species invasion, but not by urban development. Invaded communities had higher community‐weighted mean body size and the feeding guild composition of invaded arthropod communities was characterized by the exceptional numbers of nectarivores, herbivores, and detritivores. With the exception of species richness and exponential Shannon diversity, invasion influenced four out of six response variables to a greater degree than urban development did. Hence, we can conclude that species invasion is not just a passenger of urban development but also a driver of change.

## Graphical abstract

This study assessed if two global, often co‐occurring threats to biodiversity—urban development and plant invasion—affect arthropod community composition in similar ways and to a similar extent. We detected that both factors influence arthropod communities, but do so differently. Urban development affected the taxonomic composition of arthropod communities through changes in species richness and diversity, while plant invasion increased arthropod abundance and caused significant taxonomic and functional biotic homogenization.

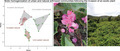

## 1 INTRODUCTION

Global change is reassembling biotic communities at exceptional rates (Capinha, Essl, Seebens, Moser, & Pereira, 2015; Ceballos, Ehrlich, & Dirzo, 2017), and two of the major causes of biotic change are urban development of natural habitats and the spread of invasive species (Clavero & Garciaberthou, 2005; Galiana, Lurgi, Montoya, & López, 2014). These two disturbances often co‐occur (Catford et al., 2012; Macdougall et al., 2014), as the development of an area makes it more prone to species invasion (Jesse, Behm, Helmus, & Ellers, 2018; McKinney, 2006). Because of this co‐occurrence, the independent effects of development and species invasion on biotic communities are largely unknown, and it is still an open question whether these disturbances change biotic communities in the same direction and to a similar extent.

Urban development introduces man‐made, impervious surfaces and substrates to ecosystems, altering microclimates through changes in temperature, humidity, and light regimes (Pickett et al., 2001). These abiotic changes directly affect the diversity, structure, and functional composition of natural communities. Vertebrate and invertebrate richness and abundance generally decrease with various levels of urban development, though results vary markedly (Faeth, Bang, & Saari, 2011; McKinney, 2008; Newbold et al., 2015). For example, arthropods seem less sensitive to development as they can maintain richness levels and even increase in abundance in urban cores, possibly because they utilize novel food sources and refugia within developed areas (Faeth et al., 2011). Urban development also changes the functional trait composition of urban biota, causing significant functional homogenization by consistently favoring generalist over specialist species (Devictor et al., 2008; McKinney, 2006). Furthermore, arthropod body size distributions change along urbanization gradients, though not for all taxa in the same direction. Generally, urban areas select for smaller species, but for large, mobile taxa, community‐level body size increases along urbanization gradients, which could greatly impact ecosystem processes, such as primary productivity, carbon cycling, and decomposition (Merckx et al., 2018).

Another major anthropogenic impact that can change species diversity and community composition is the establishment and invasive spread of exotic species (Hooper et al., 2005; Vilà et al., 2011). Invasive plant species in particular can disrupt native communities through several mechanisms. First, exotic plants can outcompete native flora for nutrients, space, and light, often decreasing the richness and abundance of native plant species (Castro‐Díez, Pauchard, Traveset, & Vilà, 2016; Michelan, Thomaz, Mormul, & Carvalho, 2010). Second, invasive plants can affect higher trophic levels within the local community, such as herbivorous arthropods, through changes in their food supply and quality (e.g., Štrobl et al., 2019) or modification of the local vegetation structure and microclimatic niches (Gerber et al., 2008; Litt, Cord, Fulbright, & Schuster, 2014; Valtonen, Jantunen, & Saarinen, 2006). Similar to urban development, invasive plants can drive functional homogenization of the vegetation (Castro‐Díez et al., 2016) as well as arthropod communities (Florencio, Cardoso, Lobo, Azevedo, & Borges, 2013). For instance, both generalist and specialist herbivores (Graves & Shapiro, 2003), detritivores (Gratton & Denno, 2005), and pollinators (Bartomeus, Vilà, & Santamaría, 2008) have been observed to extend or switch their diet and behavior to exploit an exotic plant species, which subsequently changes the arthropod community from being dominated by specialized species that have coevolved with native flora to communities containing generalist and highly adaptable species that perform similar functions (Harvey & Fortuna, 2012).

Development and invasion may act simultaneously, as the first point of introduction and establishment of exotic species is often in developed areas (Kowarik, 2011). Exotic species generally have wider ecophysiological tolerances and plasticity in resource acquisition compared to native species (Funk & Vitousek, 2007), giving them a strong competitive advantage in human‐impacted ecosystems (Sax & Brown, 2000; Shea & Chesson, 2002). As a consequence, successfully invading species benefit disproportionately from the development of natural landscapes and are mostly found in anthropogenic habitats, such as managed gardens, suburban areas, and ruderal habitats (Jesse et al., 2018; McKinney, 2008). Hence, where invasive species are studied in developed areas, the effects of development and invasion are intertwined, obscuring the causes and mechanisms that underlie the observed biodiversity patterns, and thereby complicating the development of effective remediation strategies. This study was designed to disentangle the individual and interactive effects of urban development and an invasive plant species on terrestrial arthropod communities.

**FIGURE 1:**
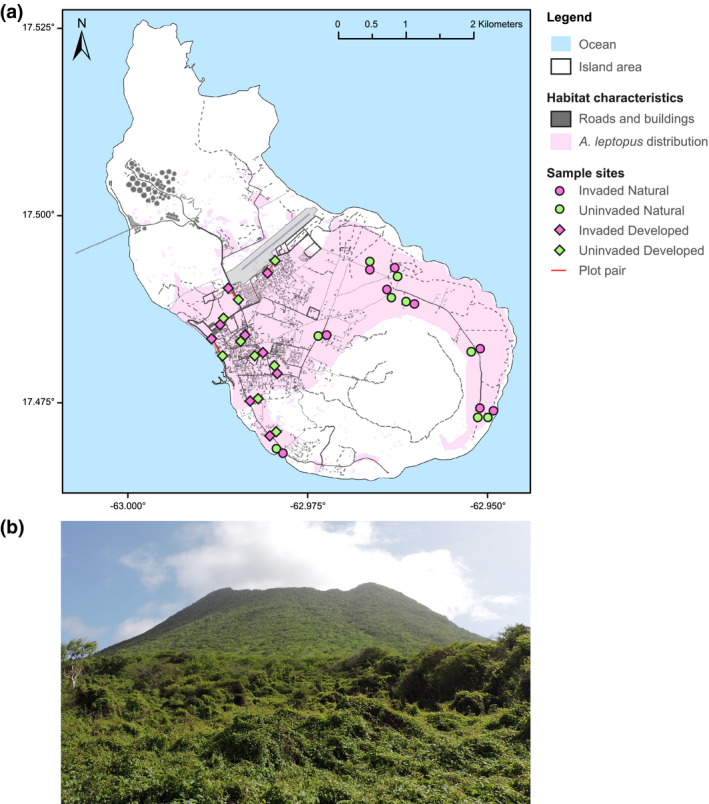
Map of 36 sample plots on the island of St. Eustatius, half of which were invaded by *Antigonon leptopus*. (a) Sampling occurred in paired locations, including an *A. leptopus*‐invaded plot (pink) and a proximate uninvaded plot (green; connected with red line in case of distantly positioned paired plots), situated widely across the introduced range of *A. leptopus* (light pink layer; edited from Berkowitz, 2014). Developed plots (diamonds) were located in areas with high levels of development including buildings and roads (layer edited from ©OpenStreetMap; OpenStreetMap contributors, 2017), and natural plots (circles) were located outside of suburban areas. The map is projected in WGS1984 coordinate system with latitude (*x*‐axis) and longitude values (*y*‐axis) presented in decimal degrees format. (b) Picture of a “Natural Invaded” sample site on St. Eustatius, situated on the eastern side of the dormant volcano “the Quill”

Here, we study the effect of the invasive plant species *Antigonon leptopus* (Hooker & Arnott, 1838) on arthropod communities in variously developed and natural habitats on the Caribbean island of St. Eustatius (Figure 1). This highly invasive species throughout the tropics (Vandebroek et al., 2018), commonly known as the Mexican creeping vine (Heger & van Andel, 2019), forms thick blankets of plant biomass (Figure 1b) thereby reducing plant diversity and impeding animal dispersal, foraging, and nesting behavior (Burke & DiTommaso, 2011). Especially on tropical oceanic islands, which are relatively species‐poor but considered hotspots for endemic species (Myers, Mittermeier, Mittermeier, Fonseca, & Kent, 2000), such disruption of ecosystem processes could have significant consequences for local biodiversity. On St. Eustatius, *A. leptopus* was the dominant plant species (>50% coverage) in 3% of the island's surface area in 2014 (Haber et al., submitted), and occurred as subdominant species in an additional 30% of the island (Figure 1a; Berkowitz, 2014). *A. leptopus* has invaded a wide variety of habitats, including developed areas, that is suburban sites (cf. rural–urban gradient by Alberti, 2015) as well as natural areas. Therefore, this is an excellent study system to investigate the effects of species invasion independent from development. We focus our study on arthropods, because they are relatively diverse and abundant, and arthropods are part of many trophic and non‐trophic interactions with local flora, for instance for their food supply (Forister et al., 2015) and microhabitat use (Johnson & Agrawal, 2005).

In this study, we test if exotic plant invasion and urban development affect (a) the taxonomic composition of arthropod assemblages, specifically species richness, total abundance, exponential Shannon diversity, and species composition; and (b) the functional composition of arthropod assemblages, specifically their community‐weighted mean (CWM) body size and feeding guild composition. We expect development and *A. leptopus* invasion to have similar effects on taxonomic composition and diversity indices, either increasing diversity by providing novel niches for generalist arthropods or decreasing diversity through the loss of specialists. We expect the feeding guild composition of invaded communities to change due to the novel food sources that *A. leptopus* provides, with higher abundances of nectarivores and detritivores, and possibly herbivorous species if *A. leptopus* proves palatable. Because nectarivores (e.g., bees and butterflies) are relatively large‐bodied, we expect additive and positive effects of development and invasion on CWM body size. Our work will elucidate whether urban development or plant invasion is more important in shaping arthropod communities in human‐impacted environments, and whether these stressors mitigate or exacerbate each other's effects.

## 2 METHODS

### 2.1 Study system

The Caribbean island of St. Eustatius is part of the Northern Lesser Antilles and has a surface area of approximately 21 km^2^. The island has a tropical climate with seasonal variation in rainfall, and the dominant vegetation consists predominantly of xeric, secondary shrubland, and woodland, with a small area of primary, evergreen forest on the dormant volcano “the Quill” on the southern side of the island (Figure 1b; de Freitas, Rojer, Nijhof, & Debrot, 2014; Rojer, 1997; van Andel, Hoorn, Stech, Arostegui, & Miller, 2016). Low‐density developed, suburban areas occupy approximately 30% of the island (Figure 1a) characterized by paved roads, detached buildings, fences, and lots of building materials. The current extent of agricultural land use is minimal (Dienst Landelijk Gebied, 2011; van Andel et al., 2016).

The native plant community of St. Eustatius is highly diverse, including ferns, trees, shrubs, and vine‐like plants, such as the endemic Statia morning glory (*Ipomoea sphenophylla*, Urb; Axelrod, 2017). *A. leptopus* is thus not the only vine species on the island, but possesses a combination of traits that make it exceptionally invasive and persistent (Burke & DiTommasso, 2011; Heger & van Andel, 2019). For instance, *A. leptopus* exhibits both sexual and clonal reproduction and grows extraordinarily fast (Raju, Raju, Victor, & Naidu, 2001), enabling it to creep and climb over various substrates in the landscape to form thick blankets of plant matter and litter underneath (Figure 1b; Ernst & Ketner, 2007). Furthermore, the plant produces tuberous roots from which the species can resprout after aboveground removal, and has a high tolerance for drought and degraded soils (Heger & van Andel, 2019). The earliest record of the exotic vine *A. leptopus* on St. Eustatius is from 1907, when it was likely introduced as an ornamental plant and feed for livestock (Boldingh, 1909; de Freitas et al., 2014). However, the free‐roaming livestock on St. Eustatius generally do not eat *A. leptopus* and prefer grazing on the native flora (Ernst & Ketner, 2007; Weiss, Muir, & Godfrey, 2010), which perpetuates its further invasive spread by disturbing the soil and reducing native plant diversity (Heger & Andel, 2019). *A. leptopus* is still absent from places of high native plant diversity and dense canopy cover such as the tropical forest on the Quill, but more open natural environments such as shrubland, open secondary woodland, and grasslands have already been invaded by the plant (van Andel et al., 2016).

### 2.2 Sampling design

To test the effect of a plant invasion on arthropod communities, we applied a paired sampling design across the entire range of *A. leptopus* on St. Eustatius (Figure 1a). Every pair consisted of an *A. leptopus*‐dominated plot (at least 80% surface coverage) and a nearby (max distance of 300 m) uninvaded control plot (*A. leptopus* absent). Plot pairs were established in both developed and natural environments. Developed plot pairs were always located in suburban areas, and included or were immediately surrounded by man‐made substrates and structures, such as pavement, houses, roads, and building materials. Natural plot pairs were located in shrubland, open woodland, or grassland, and devoid of development. Some natural plots were placed in former farmland that was recolonized by pioneer vegetation after having been abandoned sometime between 1960 and the early 1990s, in which the invasion of *A. leptopus* has ceased the process of natural succession (Ernst & Ketner, 2007; van Andel et al., 2016). Eighteen plot pairs were sampled between 3 March 2016 and 28 April 2016 (nine pairs in developed and nine pairs in natural habitat, 36 plots in total). Plots were 10 m × 8 m, and plots of the same pair were similar in elevation and slope. Plot pairs were always sampled on the same date and for the same duration, with exception of the first pair that was sampled on two consecutive days with similar weather conditions. See Appendix S1, Figure S1 for a schematic overview of the sampling design.

### 2.3 Arthropod sampling

Three different sampling techniques were employed to sample arthropod diversity in the plots. First, 50% of the plot surface area was sampled using a standard sweep net, making one sweep through the top of the vegetation with every step. The sweep net was submerged in water and all arthropods (≥0.5 mm) were collected and preserved in 70% ethanol. Second, in the other plot half, all bees and butterflies were recorded by walking along the long edge of the plot and pausing at every 2 m × 4 m portion to observe it for the duration of 1 min, amounting to a total observation time of 5 min. We employed this “observational” method a minimum of 10 min after sweep netting had occurred to reduce any disturbing effects of sweep net sampling on observational sampling. Third, in the same plot half where the observational survey was completed, five yellow pan traps (16 cm diameter, 5 cm depth) were placed approximately 50 cm above the ground to trap pollinator species (Cane, Minckley, & Kervin, 2001; Vrdoljak & Samways, 2012). Hot sauce was added to the pan rims, to prevent free‐roaming livestock from drinking the water. Pan traps were placed in the morning (between 9:00 and 10:00 hr) and removed after 8 hr (between 17:00 and 18:00 hr). We were unable to sample ground‐dwelling species, as we could not use pitfall traps due to the possibility of harming protected non‐target species (e.g., endemic reptiles). Also, a commonly used alternative for pitfalls, performing ground observations, was not possible in a standardized manner because of the disturbing effects of removing the thick litter layer in *A. leptopus*‐invaded sites. Our results are therefore constrained to compositional changes in diurnal flying insects and arthropods living in the vegetation, rather than on the ground. See Appendix S1, Figure S2 for an overview of the plot dimensions and use of sampling methods therein.

We recorded weather conditions, sampling date, and flower density in the plot to include as covariables in our statistical models. Weather conditions were recorded on‐site during sweep net and observational surveys, and daily weather conditions during pan trap sampling were determined based on data obtained from a local weather station (S. Works, personal communication, March 25, 2018) and archived satellite images (Royal Netherlands Meteorological Institute, 2016). Cloud cover was divided into three ordered categories (1 = no clouds, 2 = partial cover, and 3 = full cover); rainfall was scored as the presence/absence (0 = no rain and 1 = rainfall), and wind speed was divided into four ordered categories (0 = no wind, 1 = 1Bft, 2 = 2Bft, and 3 = 3–4 Bft; see Appendix S1, Figure S3 for more information). We did not sample in conditions exceeding maximum wind speeds. Flower densities were converted to an ordered numerical index (0 = no flowers, 1 = ~5 flowers/m^2^, and 2 = ~20/m^2^).

### 2.4 Species identification, feeding guild allocation, and body size measurements

All sampled arthropods were identified to species level when possible. However, the arthropod fauna of St. Eustatius is poorly described, which meant that we relied on morphospecies for a substantial portion of the collected material. We identified specimens by eye to morphospecies (hereafter referred to as ‘species’), based on the most detailed taxonomic level possible (usually family or superfamily level: e.g., “Diptera Dolichopodidae species 1,” “Hemiptera Aphidoidea species 2”). We subsequently assigned each species to one of the six feeding guilds: herbivores, nectarivores, predators, parasitoids, detritivores, and omnivores (see Appendix S1, S4 for detailed descriptions). Feeding guilds reflect the dominant feeding strategy within the respective taxon, according to various field guides and scientific literature (see Appendix S2, Table S1 for references). In addition, we measured the body size of all individuals from sweep net and pan trap samples in millimeters from the tip of the head to the end of the abdomen. The visually observed bee and butterfly species were assigned a mean body size based on caught individuals of the same species within sweep net and pan trap samples or data from the literature (Appendix S2, Table S2).

### 2.5 Statistical analysis

#### 2.5.1 Arthropod richness, abundance, exponential Shannon diversity, and body size distribution

We calculated arthropod species richness, total abundance, exponential Shannon diversity, and CWM body size of arthropods for every sampling method (i.e., sweep net, pan traps, and observational surveys) per plot to be able to account statistically for the effect of our selected sampling methods on these variables. The exponential Shannon diversity index (exp. Shannon), maximized when communities are species rich and relative abundances are even across species (Chao, Chiu, & Jost, 2014; Hill, 1973), was calculated by taking the exponent of classic Shannon diversity values calculated with the “diversity” function (“vegan” package; Oksanen et al., 2016). The benefit of using exp. Shannon as diversity metric is that it increases linearly with community complexity, while classic Shannon does not, preventing the data from being positively skewed toward high, and more even diversity estimates (Chao et al., 2014). CWM body size was calculated by including species frequencies as weights in the “wtd.mean” function (“Hmisc” R package; Harrell et al., 2019).

Dependent variables were included in separate linear mixed models (“lmer” package; Bates, Mächler, Bolker, & Walker, 2014) to test against the effects of invasion (invaded vs. uninvaded), development (developed vs. natural), and their interaction, as well as sampling date, flower density, cloud cover, rainfall, and wind speed as covariables. In addition, we added two categorical random effects to the model: sampling method, which accounted for variation in arthropod yield between our selected sampling methods, and sampling plot nested in plot pair to correct for interdependence among samples and potential site‐specific effects on the variables of interest. All possible models from the combination of the fixed variables were generated using the “dredge” function in the “MuMIn” package (Barton, 2018), and all models within two units of corrected AIC from the model with lowest AICc value (ΔAICc < 2) were averaged with the “model.avg” function (“MuMIn”). We tested for independence and normality of residuals in all resultant averaged models and dependent variables were either square root (i.e., species richness and exponential Shannon) or natural log transformed (i.e., total abundance and CWM body size) to achieve the best model fits. The selected independent variables in the averaged models were checked for collinearity using the variance inflation factor (VIF) with a threshold value of 2 (“car” package; Fox & Weisberg, 2018). To ensure that differences in observed species richness were not a result of sampling error, we compared observed species richness data with two estimates of true species richness per sampling method per plot (i.e., expected species richness with unlimited sampling). We calculated true species richness with Hurlbert's rarefaction analysis (Hurlbert, 1971) and non‐parametric Chao estimation (Chao, 1987), using the “rarefy” and “estimateR” functions in the “vegan” package (Appendix S1, Figure S5).

#### 2.5.2 Taxonomic and feeding guild community composition

We used a partial redundancy analysis (pRDA) provided by the “vegan” package to test whether differences in arthropod community composition between plots were associated with *A. leptopus* invasion, urban development, and their interaction. We grouped the three sampling methods to represent complete arthropod communities per plot, and applied a Hellinger transformation on the resultant species‐abundance matrix (185 species × 36 plots). This transformation reduces the impact of highly abundant species on the results, thereby making the community score per sample more representative of the full community (Borcard, Gillet, & Legendre, & Legendre, P., 2011). In addition to our focal factors, we included flower density, and the mean values of the rain, wind, and cloud cover indices per plot as constraints in the pRDA model to exclude any community variation attributable to these covariables. We did not include sampling date as a covariable in this model, because it was negatively related to mean wind speed (*r* = −.79, *df* = 34, *t* = −7.45, *p* < .001). Variation in feeding guild composition among plots was assessed in a similar fashion as taxonomic species composition. All species per plot were grouped into their appointed feeding guild and included as Hellinger‐transformed matrix into a pRDA that accounted for variation in mean weather conditions and flower density.

To test validity of our results, we first confirmed that there was no spatial autocorrelation of the pRDA model residuals (“mso” function; “vegan” package), ensuring that we did not have to account for sample location in the model (Appendix S1, Figure S6). In addition, we checked whether our results were robust against differences in taxonomic resolution (i.e., the level to which species could be identified) by grouping arthropods into higher taxa and rerunning the ordination analyses (Appendix S1, Table S7). Lastly, we assessed whether the observed compositional differences in the complete arthropod communities were consistent across the subcommunities yielded by the three sampling methods, or whether a specific sampling method biased the overall outcome (Appendix S1, Figure S8).

## 3 RESULTS

### 3.1 Taxonomic diversity and species composition

In total, 4,690 arthropods were collected or observed in the field, of which 1,597 were caught in pan traps, 2,518 through sweep netting, and 575 in observational surveys. The three sampling techniques yielded a total of 185 species divided over 11 arthropod orders. See Appendix S1, Figure S9 for a detailed overview of sampled arthropods.

**FIGURE 2:**
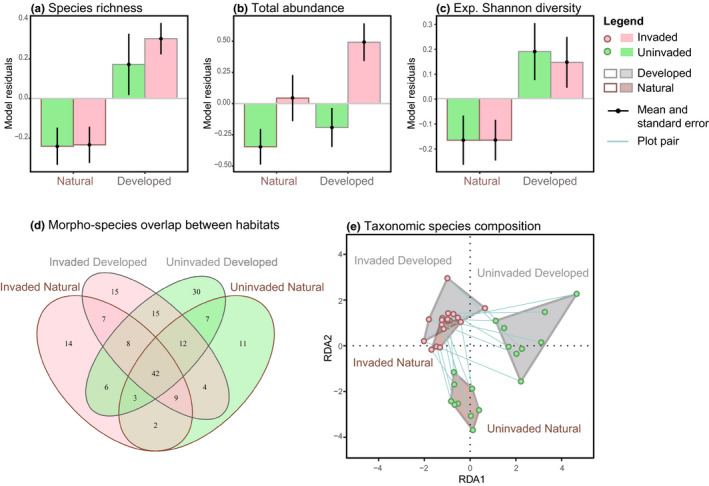
Taxonomic diversity and species composition as a function of exotic plant invasion and urban development. Effects of development and *Antigonon leptopus* invasion on (a) arthropod species richness, (b) arthropod total abundance, and (c) exponential Shannon diversity per sample. Plotted values are model residuals from the model‐averaged linear mixed effects models depicted in Table 1, excluding fixed effects of development and invasion. (d) Venn diagram of species overlap (total = 185 species) among the four invasion‐development habitat types in our study: *Invaded Natural*, *Uninvaded Natural*, *Invaded Developed*, and *Uninvaded Developed*. (e) Differences in taxonomic species composition between sampled plots. Points are colored according to invasion category (invaded: pink, uninvaded: green) and convex hulls around the four invasion‐development habitat types are colored according to the development category (developed: grey, natural: brown). Pairwise distances between points represent the relative differences in species‐abundance composition between communities

Arthropod species richness per sampling method was significantly higher in developed plots compared to natural plots (*z* = 3.19, *p* = .001; Table 1; Figure 2a), amounting to a mean increase of 8.8 species per plot, cumulative over the different sampling methods (33% of mean plot species richness). Across all samples, a total of 60 species (32% of all sampled species) were only found in developed plots, in contrast to 27 unique species in natural plots (Figure 2d). In addition, there was a positive effect of flower density on species richness (*z* = 2.04, *p = *.04; Table 1). Even though we did not detect a significant effect of *A. leptopus* invasion on observed species richness, rarefied species richness was significantly higher in pan trap and observational samples in *A. leptopus*‐invaded sites (non‐parametric Scheirer–Ray–Hare: *H*_1,35_ = 11.9, *p* < .001 and *H*_1,35_ = 8.5, *p* = .004, respectively; Appendix S1, Figure S5), indicating that true species richness could have been higher with more vigorous sampling. However, no such relationship was detected for Chao‐estimated (i.e., extrapolated) species richness.

**TABLE 1:** Results of model‐averaging procedure to explain arthropod species richness, total abundance, exponential Shannon diversity, and the CWM body size per sampling method. The table depicts the model coefficients of only those variables that were included in models within two units of corrected AIC (ΔAICc < 2) from the top‐ranking model to explain their respective dependent variable (italic). The number of top‐ranking models involved in model‐averaging is depicted in brackets next to the focal dependent variable. The importance of a variable to explain the focal dependent variable is expressed as sum of its Akaike weights over all top‐ranking models that the variables appears in, amounting to maximum value of 1.00 if it appeared in all models. Statistically significant *p*‐values (*α* < 0.05) are shown in bold

Selected variables in top‐ranking models (ΔAICc < 2)	Model‐averaged coefficients				Variable importance (Σ Akaike weights)
	Estimate	(Adj.) *SE*	*z*	*p*	
*Species richness* ^a^ (2 models)					
Development	0.48	0.15	3.19	**<.01**	1.00
Flower density	0.20	0.10	2.04	**.04**	0.37
*Abundance* ^b^ (2 models)					
Development	0.31	0.16	1.87	.06	0.43
*Antigonon leptopus* invasion	0.56	0.17	3.33	**<.001**	1.00
Flower density	0.31	0.12	2.69	**<.01**	1.00
*Exponential Shannon diversity* ^a^ (3 models)					
Development	0.33	0.13	2.58	**<.01**	0.77
Rainfall	0.30	0.16	1.87	.06	0.31
*CWM body size* ^b^ (3 models)					
*A. leptopus* invasion	0.16	0.05	2.98	**<.01**	0.79
Rainfall	−0.18	0.10	1.84	.07	0.24

^a^ Square root transformation.

^b^ Natural log transformation.

Arthropod abundance per sampling method was not significantly affected by urban development (*z* = 1.87, *p* = .06), but increased with *A. leptopus* invasion (*z* = 3.33, *p* < .001; Table 1; Figure 2b), totaling a cumulative mean increase of 51.7 individuals in invaded plots (39% of the mean arthropod abundance per plot). Arthropod abundances further increased with increasing flower densities (*z* = 2.69, *p = *.007). Even though *A. leptopus* can form dense flowerbeds, *A. leptopus* invasion and flower density were not significantly related (0.28 ± 0.24, *t* = 1.14, *p* = .26), and independently affected arthropod abundance (VIF < 1.5).

Exponential Shannon diversity was significantly higher in developed plots compared to natural plots (*z* = 2.58 *p* = .0099), but was unaffected by *A. leptopus* invasion (Table 1; Figure 2c). Arthropod communities in developed plots were thus more compositionally complex than in natural plots, which resembled our outcome for species richness (Table 1; Figure 2a,c). One out of the three top‐ranking models included rainfall as a positive predictor for arthropod diversity; however, this effect was not statistically significant (*z* = 1.87, *p* = .06).

Arthropod species composition differed significantly between invaded and uninvaded plots (*F*_1,35_ = 1.54, *p* < .001) and between plots in natural and developed plots (*F*_1,35_ = 1.41, *p* = .002; Figure 2e). The species composition of invaded developed and invaded natural plots partially overlapped, whereas uninvaded plots occupied distinct portions of the ordination space (Figure 2e). There was no significant interaction between development and invasion, suggesting that there was no difference in the direction in which natural and developed communities shifted in the pRDA ordination space following *A. leptopus* invasion (*F*_1,35_ = 1.18, *p* = .08). A total of 27% of community compositional variation was explained by the statistical model, divided over development, invasion, and their interaction (16%), as well as weather conditions and flower density (11%). These results persisted when we assessed the different levels of taxonomic resolution, as family‐level and higher taxon analyses showed similar results (Appendix S1, Table S7). Furthermore, the community‐level results were not biased by the yield of one specific sampling method, rather all parts of the community contributed to the overall observed patterns (Appendix S1, Figure S8).

### 3.2 Body size distribution and feeding guild composition

**FIGURE 3:**
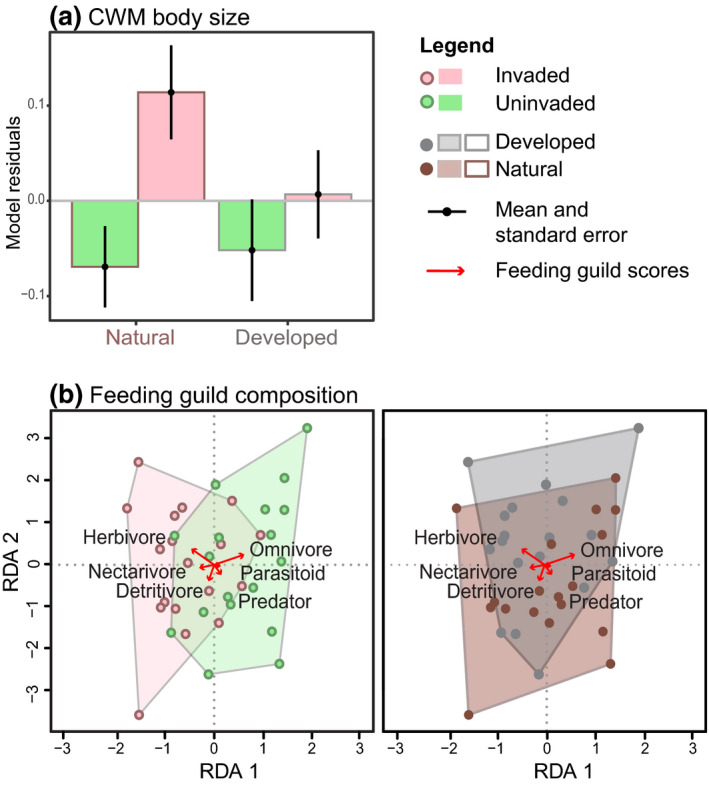
Functional community composition as a function of exotic plant invasion and urban development. (a) Effects of invasion and development on CWM body size. Values are model residuals from the model‐averaged linear mixed effects models depicted in Table 1 without the fixed effects of development and invasion. (b) Differences in feeding guild composition between invaded (pink) and non‐invaded (green) sites (b, left graph) and between natural (brown) and developed (grey) areas (b, right graph). Pairwise distances between points represent the relative differences in species‐abundance composition between communities

CWM body size per sample was positively affected by *A. leptopus* invasion (*z* = 2.98, *p* = .003), resulting in a mean size increase of 0.92 mm per sample (20% of mean arthropod body size; Table 1; Figure 3a). Only in one out of the three best‐fit models, rainfall was selected as negative predictor for CWM body size; however, this effect was not statistically significant (*z* = 1.84, *p* = .07).

Variation in feeding guild composition between plots was significantly associated with *A. leptopus* invasion (*F*_1,35_ = 2.13, *p* = .04), though compositional overlap was considerable (Figure 3b). Herbivores, nectarivores, and detritivores were particularly attracted to invaded plots, while species from omnivorous taxa were disproportionately associated with uninvaded plots. In contrast, urban development did not affect feeding guild compositions of arthropod communities (*F*_1,35_ = 1.27, *p* = .28; Figure 3b). Twenty‐six percent of compositional variation was explained by the statistical model, divided over the variables of interest and their interaction (15%), as well as weather conditions and flower density (11%).

## 4 DISCUSSION

In this study, we disentangled the effects of two globally occurring anthropogenic disturbances: urban development and species invasion. Our results show that both urban development and *A. leptopus* invasion fundamentally change arthropod communities, but urban development affected the taxonomic composition through changes in species richness and diversity, whereas plant invasion also changed the functional composition of arthropod communities. Furthermore, we detected taxonomic and functional homogenization of arthropod communities following *A. leptopus* invasion, resulting in distinct communities in *A. leptopus*‐invaded areas that are likely unique in structure and function compared to other arthropod communities on the island.

### 4.1 Taxonomic and functional responses to urban development and invasion

#### 4.1.1 Urban development

Species richness was positively affected by development, creating compositionally diverse communities compared to natural sites. Uninvaded developed plots harbored communities with distinct composition compared to uninvaded natural plots, including relatively many unique species compared to the other sampled habitat types. These results could be due to a higher availability of microhabitats and novel niche space in urban‐developed areas (Shea & Chesson, 2002), attracting more species from the local species pool (Fetridge, Ascher, & Langellotto, 2008), or alternatively, providing niche space to introduced arthropods (Borges et al., 2006; Niemelä et al., 2011). Though our results on arthropods differ from the general trend that animal diversity decreases with various levels of urban development (McKinney, 2008), they do fit into the context of the intermediate disturbance hypothesis that states that diversity peaks in the midrange of urban development (Wilkinson, 1999). Also an earlier study showed a similar pattern for reptile diversity on St. Eustatius, with the highest overall reptile diversity in developed (i.e., suburban residential) areas on the island, including the individuals of several exotic species (Jesse et al., 2018). For the current study we cannot definitively conclude that the increase in arthropod species richness in developed areas is also the result of exotic species invasion because we do not have sufficient data on species' origin. Furthermore, the fact that both arthropod richness and exponential Shannon diversity were relatively high in developed sites compared to natural sites indicates that the additional species in developed areas occur in similar abundances as other species in the community (Chao et al., 2014; see Appendix S1, S10 for further details). Overall this supports the hypothesis that the higher species richness in developed areas may have resulted from an influx of native urban adapters, rather than dominant invasive alien species that disproportionately exploit this suburban ecosystem, as has been found in several other systems (see Snyder & Evans, 2006 for examples). A caveat to our results is that the estimate of species richness was based on morphospecies, identified by the similarity of the species without robust arthropod identification keys. This may have artificially increased species counts, for example if sexually dimorphic and environmentally polymorphic species were divided into different morphospecies. To account for this possibility, three expert entomologists independently checked and verified all morphospecies records based on photographic data, which resulted in a minor reduction of three out of 188 species through conservative reassignment of morphospecies. Furthermore, our species richness estimates did not include ground‐dwelling species, and thus is an underestimation of the total species richness, in which particularly the Hymenoptera and Diptera may be overrepresented as opposed to, for instance Coleoptera (Appendix S1, Figure S9). Therefore, our conclusions only apply to the effects of invasion and development on mobile, diurnal species.

#### 4.1.2 Antigonon leptopus invasion

Uninvaded communities showed a clear difference in species composition between developed and natural sites, but when invaded by *A. leptopus*, communities in both environments were highly similar, with a completely novel species composition. Invaded communities had substantially higher arthropod abundance and equal species richness levels compared to uninvaded sites. These results were in line with a meta‐analysis by Fletcher et al. (2019), who detected no significant negative effects of invasive plants species on either native or exotic arthropods, as opposed to Litt et al. (2014) who concluded that a majority of arthropod studies report decreasing richness and abundance levels following plant invasion. The detected increase in arthropod abundance and absence of a negative effect on species richness in this study may be explained by the extremely high standing biomass that *A. leptopus* produces (Ernst & Ketner, 2007) which may provide arthropods with more and novel microclimatic niches, in which temperature and humidity regimes could favor species from particular feeding guilds or arthropods in general (de Groot, Kleijn, & Jogan, 2007). Given the small distance between paired invaded and uninvaded plots (10–300 m) and the fact that our sampling techniques favored highly mobile, diurnal flying arthropods, we assume that species will have been able to move between plots. Therefore, dispersal may be a homogenizing factor for the sampled arthropod communities, especially within plot pairs. The fact that we still detected consistent and clear compositional differences between invaded and uninvaded spatially paired communities therefore provides strong evidence that *A. leptopus* is a major driver of local biodiversity change in this study system.

Invaded communities included relatively high proportions of nectarivores, herbivores, and detritivores. Nectarivorous arthropods likely profit from the dense flowerbeds that *A. leptopus* produces, which bloom year‐round (Ernst & Ketner, 2007) and provide a continuous supply of nectar (Barth, 2005). This attracted many pollinating bees, butterflies, and hoverflies to invaded areas, even when plots had relatively low flower densities. These results are consistent with previous studies that revealed when the invasive plant is a pollination generalist (native) nectarivores can be tempted to pollinate the invader (Bartomeus et al., 2008; Picanço, Gil, Rigal, & Borges, 2017; Traveset & Richardson, 2006). While we anticipated *A. leptopus* to disrupt obligate plant–herbivore interactions and thus cause a reduction in herbivore abundance (e.g., Hartley, Rogers, & Siemann, 2010), we observed an unexpected increase in herbivore abundance. This suggests that herbivores may be feeding on *A. leptopus* and thus successfully incorporated *A. leptopus* into the local food web. The relatively high detritivore abundance in invaded plots is most likely due to the volume of decaying plant biomass, including *A. leptopus* litter as well as the remains of smothered, prior vegetation. The high abundance of detritivores in the community may increase local rates of decomposition and nitrogen and carbon cycling (Allison & Vitousek, 2004), potentially facilitating the further invasive spread of *A. leptopus* (e.g., Kaproth, Eppinga, & Molofsky, 2013). *A. leptopus* thus stimulates feeding guilds that can directly benefit from the invasive plant as food source as opposed to, for instance predatory taxa, which suggest that generalist species from these taxa have managed to incorporate *A. leptopus* into their diet. Interestingly, taxa indicated as omnivorous (e.g., ants) that seem intrinsically generalistic appear to primarily suffer from *A. leptopus* invasion and may be particularly sensitive to the habitat structural effects of *A. leptopus* (Lenda, Witek, Skórka, Moroń, & Woyciechowski, 2013).

The differences in community composition between invaded and uninvaded plots were accompanied by a significant increase in CWM body size in invaded plots. Nectarivores, predators, parasitoids, and omnivores all increased in CWM body size, which can largely be attributed to increase in relative abundance of large‐bodied species rather than an influx of new species, or intraspecific size increases following *A. leptopus* invasion (Appendix S1, Figure S11). The increased body size of predatory feeding guilds can simply be a consequence of a size increase of their prey (Raupp, Shrewsbury, & Herms, 2010). Alternatively, the overall increases in CWM body size and arthropod abundance can also be the result of reduced predation pressure by insectivorous vertebrates. Previous findings by our group indicate that increased coverage of *A. leptopus* leads to a significant decrease in the abundance of predatory lizards of the genus *Anolis* (Jesse et al., unpublished). In uninvaded habitats, these lizards are highly abundant (Jesse et al., 2018) and have a preference for large prey items, so that removal of these predators positively affects arthropod abundance (Pacala & Roughgarden, 1985) and arthropod body size on St. Eustatius (Dial & Roughgarden, 1995).

### 4.2 Wider geographic relevance and recommendations

Our results indicate that urban development and the invasion of *A. leptopus* both shape arthropod communities on St. Eustatius, but development affected only taxonomic composition through a positive effect on species richness, whereas invasion significantly influenced various aspects of taxonomic and functional composition of arthropod communities. One reason for the relatively low impact of urban development on arthropod assemblages could be the relatively low levels of urban development on St. Eustatius, where fully developed cities are absent. Therefore, the impact of urbanization may be low compared to other locations (see Faeth et al., 2011 for review). However, our results demonstrated a significant effect of development on arthropod species composition, so we can conclude that even moderately developed areas put unique selection pressures on arthropod communities. We estimate that with more intense development, the positive effect of development on species richness may diminish, as arthropod diversity may, depending on the focal taxon, peak at intermediate disturbance levels (Blair & Launer, 1997). Because of that, it may also be more difficult to quantify the effect of invasion in extremely developed urban sites due to the relatively low species richness. Some of these concerns may already be alleviated by the fact that we sampled on an oceanic island, and insular communities are generally species‐poor compared to communities on the continental mainland (Kier et al., 2009). Furthermore, given that insular species assemblages are characterized by relatively many endemic (and often specialist) species (Kier et al., 2009; Seebens et al., 2017), we expected to find exaggerated negative effects of urban development and invasion, but we detected generally positive associations between these anthropogenic impacts and diversity and biomass variables. Although we cannot simply expand the relevance of our conclusions to a global scale, we believe our results are highly relevant for similar ecosystems, as many tropical oceanic islands have been invaded by this specific plant species (Vandebroek et al., 2018). Furthermore, this study shows that while urban development is often seen as a facilitator of species invasion (e.g., MacDougall & Turkington, 2005), plant invasion has strong effects on the community composition of higher trophic levels independent of development. This is consistent with Elleriis, Pedersen, and Toft (2015) and Štrobl et al. (2019) who reported robust effects of invasive plants on paired arthropod assemblages in temperate ecosystems in Continental Europe while accounting for site‐specific environmental variation. Hence, plant invasion is not just a side effect of urban development but also an important driver of ecological change.

Our results regarding plant invasion and urban development can be explained in light of novel niches that provide more space and new resources for arthropod communities. Because of this, one might expect to find synergistic effects of urban development and plant invasion. However, we found that invasion affected taxonomic species composition in a different direction than development did, forming communities that have distinct compositions compared to their uninvaded counterparts. This suggests that the novel niches provided by developed areas to arthropod species become occupied when *A. leptopus* invades, while also providing unique ecological niches in this novel *A. leptopus*‐dominated habitat. Therefore, it may be advisable to manage an invasive plant such as *A. leptopus* in developed as well as natural ecosystems in order to conserve the entirety of taxonomic and functional variation that these ecosystems harbor. Providing green spaces in the urban matrix and restoring invaded natural habitats may help to achieve this goal. Fortunately, several projects and policies have been initiated to meet this challenge on St. Eustatius, such as an invasive species policy plan (Mitchell et al., in prep) and reforestation efforts, and we hope that the conclusions of this paper contribute to informed policy planning against *A. leptopus* on other tropical oceanic islands.

## Supporting information

Appendix S1

Appendix S2

## Data Availability

All the data featured in this research article is made publically available in data repository Figshare via https://doi.org/10.6084/m9.figshare.12000069.
