# Supplementary material for: Disentangling the effects of plant species invasion and urban development on arthropod community composition
Source: Glob Chang Biol. 2020 Apr 16;26(6):3294–306. doi: 10.1111/gcb.15091 (PMC7317202; doi:10.1111/gcb.15091)
Supplement: Supplementary file 1 — Appendix S1 [file GCB-26-3294-s001.pdf]

Appendix

S1.

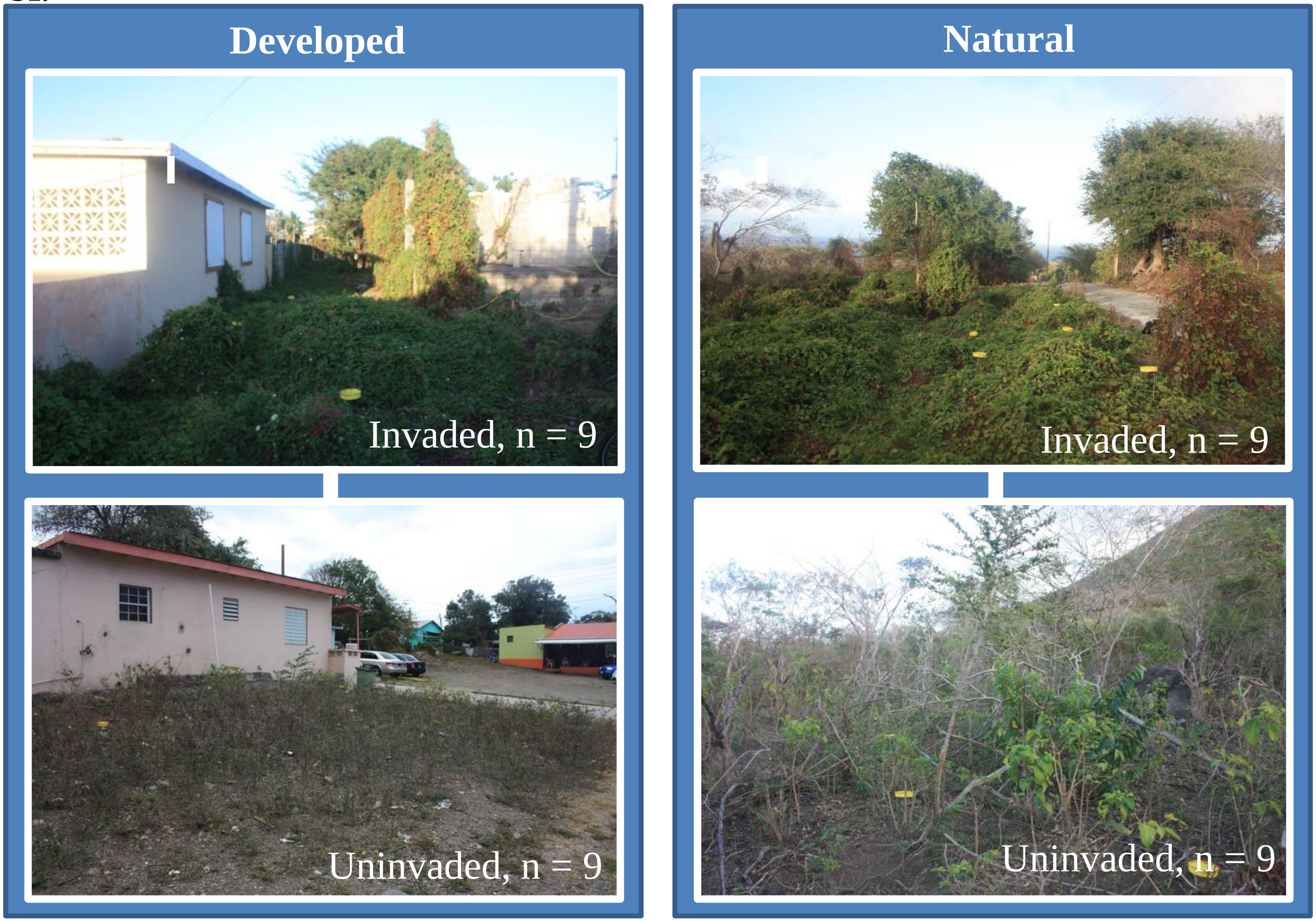

Figure S1. Overview of paired sampling design. A total of 36 plots were sampled, consisting of 18 pairs of invaded and uninvaded plots. Nine pairs were located in urban-developed areas, and 9 in natural habitats, which have distinct habitat structures.

S2.

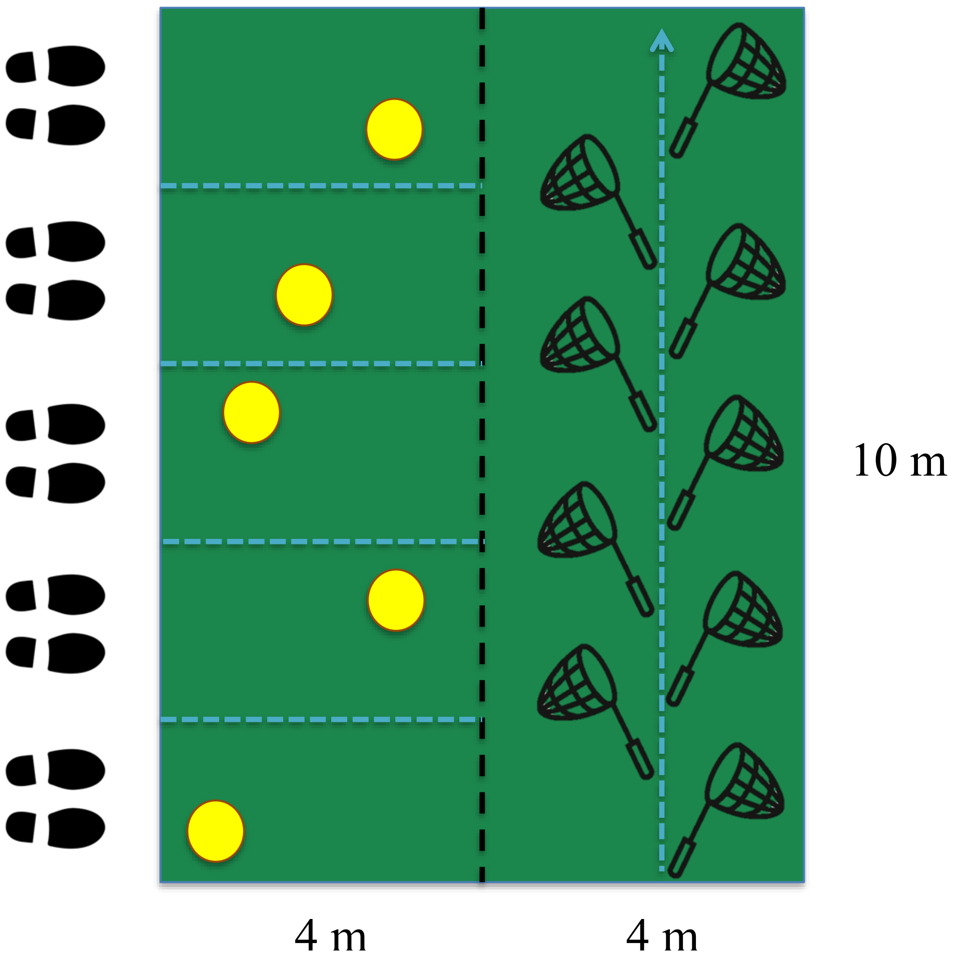

Figure S2. Overview of plot dimensions and employed sampling methods. First, half of the plot surface area (40m^2) was sampled using a sweep net, making one sweep through the top of the vegetation with every step in alternating directions from left to right (sweep net icons). All arthropods ≥ 0.5 mm body size were collected and preserved in 70% ethanol. Ten minutes after sweep netting had occurred, co-author Jasper Molleman proceeded with observational sampling in the other plot half, by walking along the long edge of the plot, and pausing every 2 m x 4 m section to record all bees and butterflies within the focal plot section for the duration of one minute (shoe icons), amounting a total observational survey time of 5 minutes. After this, pan traps were placed in the same plot half for a duration of 8 hours (yellow circles). We are confident that methodology minimizes potential repeated observations and effects of disturbance caused by sampling.

S3.

A) Level of sunshine/cloud cover

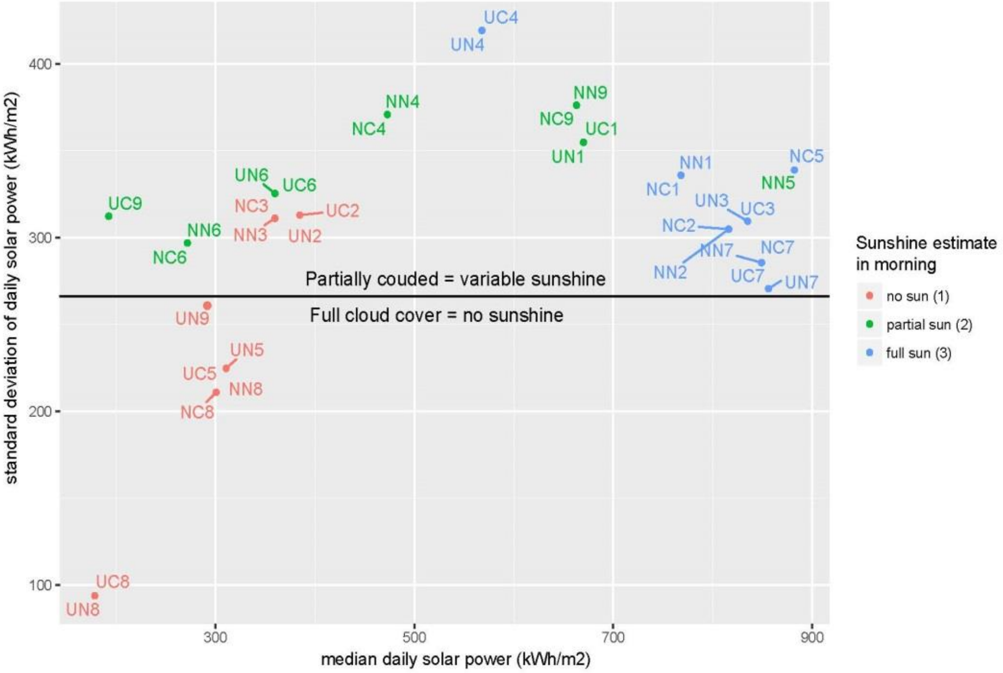

B) Wind speed conversion table

Wind force Beaufort (value)	Description	Visual representation of wind speed	Average wind speed		
			m/s	Knots	km/h
0	No wind	No rustling leaves, no noticeable wind	0.0-0.2	<1	<1
1 (1)	Gentle	Rustling leaves, wind direction would be deducible from smoke plumes	0.3-1.5	1-3	1-5
2 (2)	Gentle	Moving leaves and twigs, noticeable breeze against your skin	1.6-3.3	4-6	6-11
3 (3)	Moderate	Twigs and branches move, dust blows up	3.4-5.4	7-10	12-19
4 (3)	Moderate	Flapping clothes, hair moves	5.5-7.9	11-16	20-28
5	Semi-powerful	Blown up dust is irritating for eyes, waves on water bodies, dust bins fall over	8.0-10.7	17-21	29-38
6	Powerful	Difficult to keep hold of umbrella	10.8-13.8	22-27	39-49
7	Hard	Difficult to walk opposite to wind direction	13.9-17.1	28-33	50-61
8	Stormy	Very difficult to move	17.2-20.7	34-40	62-74
9	Storm gusts	Blowing debris, kids are unable to remain upright	20.8-24.4	41-47	75-88
10	Heavy storm	Major damage to buildings, adults unable to remain upright	24.5-28.4	48-55	89-102
11	Very heavy storm	Major damage to forests	28.5-32.6	56-63	102-117
12	Hurricane	Complete destruction	>32.6	>63	>117

Figure S3. Quality check and quantification of daily weather data during pan trap sampling. A) Daily solar level (median, x-axis) and solar variation (standard deviation, y-axis) from a local weather station were crosschecked with recorded weather circumstances during sweep net and transect sampling (colors) and with cloud estimates from a global cloud cover map (source: http://msgcpp.knmi.nl). The horizontal line indicates the border between full cloud cover and

variable cloud cover (i.e., equivalent to variable sunshine) as depicted in the global cloud cover map. This division is compatible with the median and standard deviation of solar power recorded by a local weather station between 09.00h and 18.00h. Full cloud cover has low solar power levels, relatively low variability, and exclusively “no sun” recordings during morning sampling. In contrast, partially clouded days have a higher variability of sunshine, relatively high levels of solar power, and “partial sun” and “full sun” recordings during morning sampling all fall above the line. Hence the cloud cover map is a good indicator for cloudiness/sunshine during pan trap sampling. There were no completely cloudless days during our sampling period.

B) We obtained wind speed data (measured in knots) between 9.00h and 18.00h on sampling dates from a local weather station, operated by Ms. Shelly Works. The median wind speed in knots was appointed a value (1, 2 or 3) to be comparable to the wind speed recorded during sweep net and pan trap sampling. We only sampled in gentle and moderate wind speeds (red) following descriptions from the Royal Netherlands Meteorological Institute (KNMI). The conversion table was edited from tables available here:
https://www.knmi.nl/kennis-en-datacentrum/uitleg/windschaal-van-beaufort;
http://www.weerstationuithuizermeeden.nl/Schaal-van-Beaufort.htm (Accessed August 2018).

S4. Feeding guild description

Detritivore: Species within a detritivorous taxon primarily feed on plant-derived detritus. Also species that feed on manure and fungi fall within this category.

Herbivore: Species within the taxon primarily feed on live plant matter including plant sap, as well as algae and lichen.

Nectarivore: Species feed on sugary substances, such as nectar, but also honeydew produced by aphids can be a food source.

Omnivore: Species are part of an omnivorous taxon, in which several roughly equally prevalent feeding strategies exist (both within species and between species). This means that species indicated as ‘omnivorous’ are always part of an omnivorous taxon, but may not adhere to an omnivorous diet themselves. We had to assign diet in this way because species-specific dietary information was largely unavailable.

Parasitoids (do not feed at all or very limited foraging on nectar): This category only includes adult parasitoids, which are predatory in their larval stage and switch to a limited diet of nectar after metamorphosis.

Predator: Species within a predatory taxon feed on live animals, which they actively hunt.

S5.

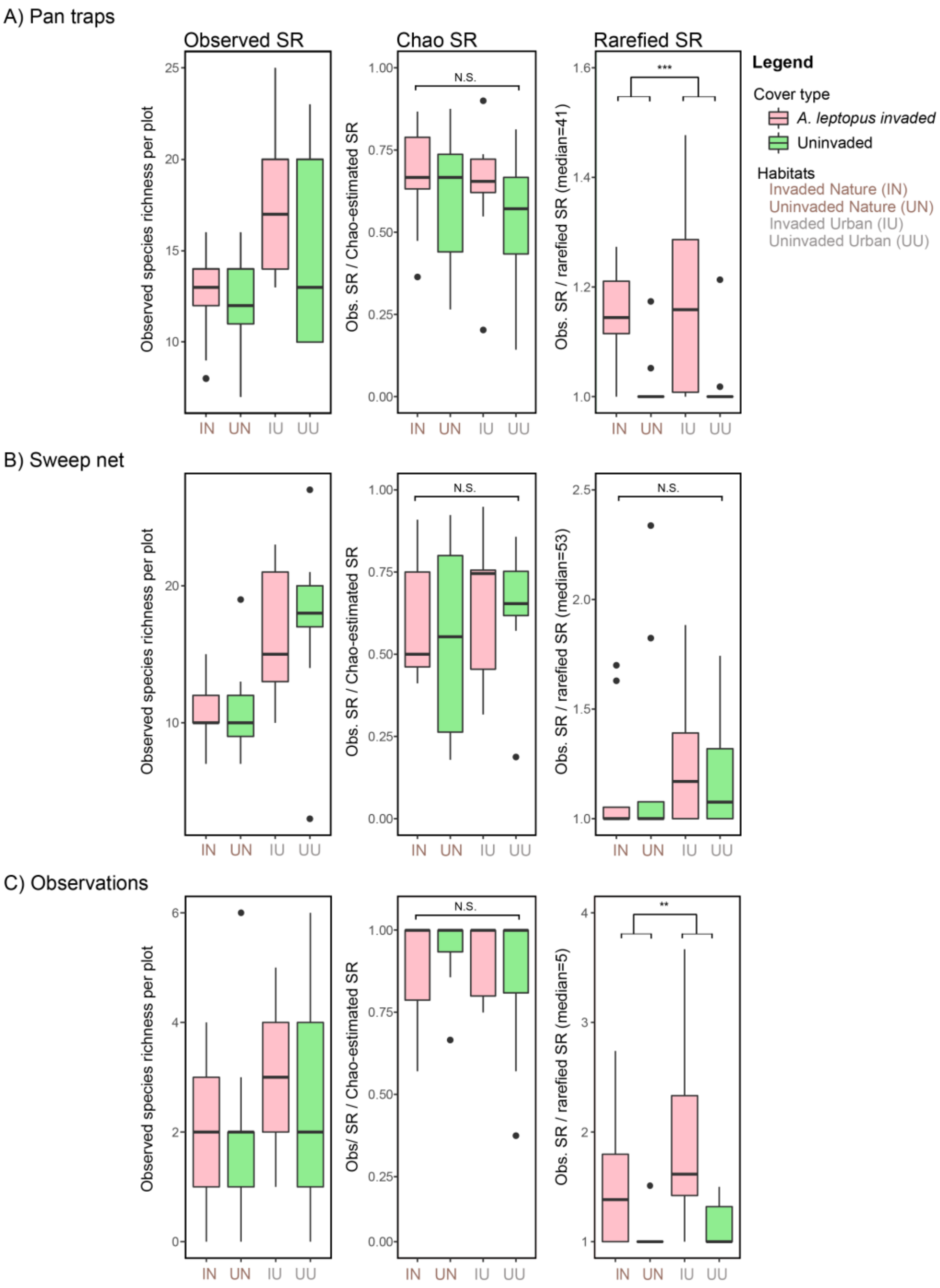

Figure S5. Completeness of sampling between invasion-development habitat categories in A) pan trap samples, B) sweep net samples, and C) observational samples. Comparison between observed species richness (left plots), and the level of sampling completeness based on two different estimates of true species richness: Chao-estimated (i.e., extrapolated richness; middle plots) and rarefied species richness (right plots), calculated with the 'estimateR' function and 'rarefy' function, respectively ('vegan' package). To calculate rarefied species richness we used the median arthropod abundance per focal method per plot as sample size.

Chao-estimated richness and corrected rarefied richness levels indicate some level of under sampling, respectively deviating negatively and positively from 1. This means that with more

vigorous sampling, we would likely have sampled more species. However, we detected no differences in Chao-estimated richness values between invasion and development categories in any of the sampling methods (indicated with 'N.S.' and tested with two-way ANOVA or non-parametric two-way Schreirer-Ray-Hare tests), indicating that observed differences in species richness represent true differences in the field. However, rarefied species richness values give some indication that pan trap (Scheirer-Ray-Hare: df=1, H=11.9, $P<0.001$) and observational samples (Scheirer-Ray-Hare: df=1, H=8.5, $P=0.004$) might be under-representations of true richness in *A. leptopus*-invaded areas. Which indicates that with more vigorous sampling, we could potentially have detected a more positive association between *A. leptopus* invasion and species richness. However, we cannot provide conclusive evidence for this possibility, given that the two estimates of true species richness yield different results.

S6.

A) Variograms of residuals of analysis on taxonomic species composition

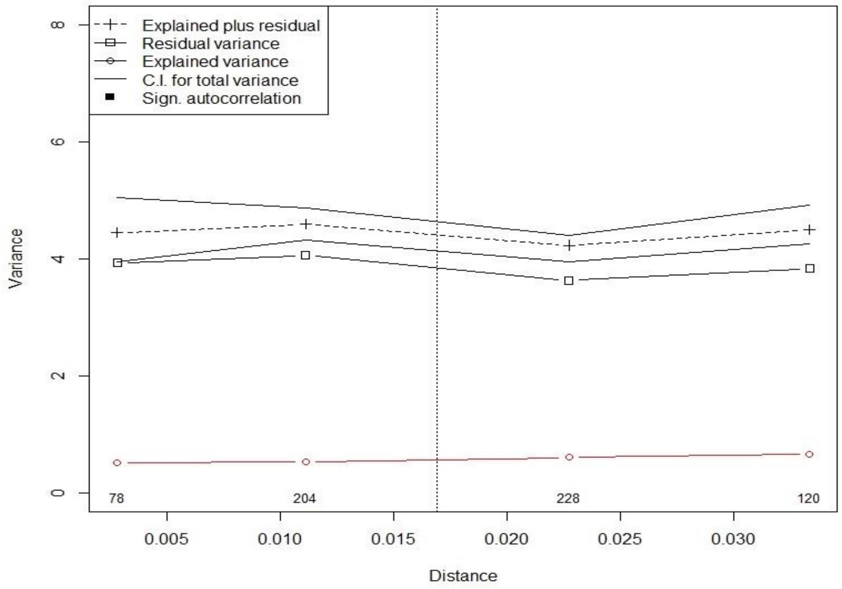

B) Variogram of residuals of analysis on feeding guild composition

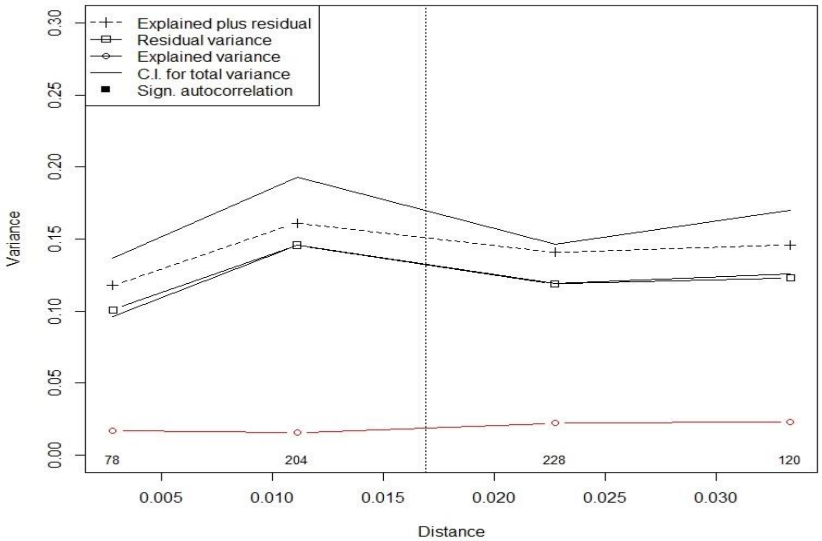

Figure S6. Spatial autocorrelation of ordination model residuals. Spatial dependency of model residuals would result in an underestimation of error variation for data points that are positioned in close proximity of each other, because they resemble each other more than expected when samples are independent replicates. Therefore, one cannot be confident in the model outcome of a partial redundancy analysis if spatial autocorrelation exist.

Such underestimation would be indicated in the variograms above as an increasing trend along a continuum of pairwise distance classes between plots (x-axis), and black rectangles to indicate significant deviation from expected and observed variation. Both are not visible for these figures, hence the effects of *A. leptopus* invasion and urban development on taxonomic species composition (A) and feeding guild composition (B) are not spatially biased.

Distance classes were automatically generated with the ‘give.thresh’ function (‘adespatial’) with an interval of 0.0113 decimal degrees ~ 1 km. P-values were corrected for multiple testing based on a false discovery rate adjustment (‘p.adjust’ function; ‘stats’).

S7.
To test whether the results were robust against changes in taxonomic resolution (i.e., the level to which species could be identified), we ran three models on community matrices in which we grouped arthropods into:

1) families, discarding all other species of lower taxonomic resolution (i.e., discarding species that could only be identified up to superfamily or order level; analysis included 89% of data)

2) unnested higher taxa: all related arthropods were grouped into the most detailed unnested thus taxonomically independent taxon (i.e., species identified up to family level (e.g., Lycaenidae) were not included in analysis with related species identified up to order level (Lepidoptera), hence all butterflies would in that case be grouped into the unnested most detailed group of Lepidoptera; analysis included 96% of data)

3) orders (including 100% of data).

Table S7. Model outputs from redundancy analyses across different levels of taxonomic resolution. The table presents the model output from the morpho-species level analysis that is featured in the main text of the article in red. Higher taxon analyses are shown below and statistically significant (α = 0.05) results are **bold**. The significant effects of development and invasion persisted when arthropods were grouped into higher taxa. The effect of *A. leptopus* invasion even nears significance when species are grouped into orders. Therefore, we can conclude that the lack of taxonomic information about the local arthropod community on St. Eustatius, and the required use of morpho-species, did not hamper us from detecting true community compositional differences associated with plant invasion and urban development.

**Taxa**	**Development**	**Invasion**	**Development*Invasion**
Morpho-species	$\mathbf{F_{1,35} = 1.4}$, $\boldsymbol{P < 0.01}$	$\mathbf{F_{1,35} = 1.5}$, $\boldsymbol{P < 0.001}$	$F_{1,35} = 1.2$, $P = 0.08$
Family	$\mathbf{F_{1,35} = 1.5}$, $\boldsymbol{P < 0.01}$	$\mathbf{F_{1,35} = 1.7}$, $\boldsymbol{P < 0.001}$	$F_{1,35} = 1.1$, $P = 0.27$
Unnested higher taxon	$\mathbf{F_{1,35} = 1.5}$, $\boldsymbol{P < 0.01}$	$\mathbf{F_{1,35} = 1.8}$, $\boldsymbol{P < 0.001}$	$F_{1,35} = 1.1$, $P = 0.27$
Order	$F_{1,35} = 1.0$, $P = 0.48$	$F_{1,35} = 1.7$, $P = 0.06$	$F_{1,35} = 0.6$, $P = 0.79$

S8.

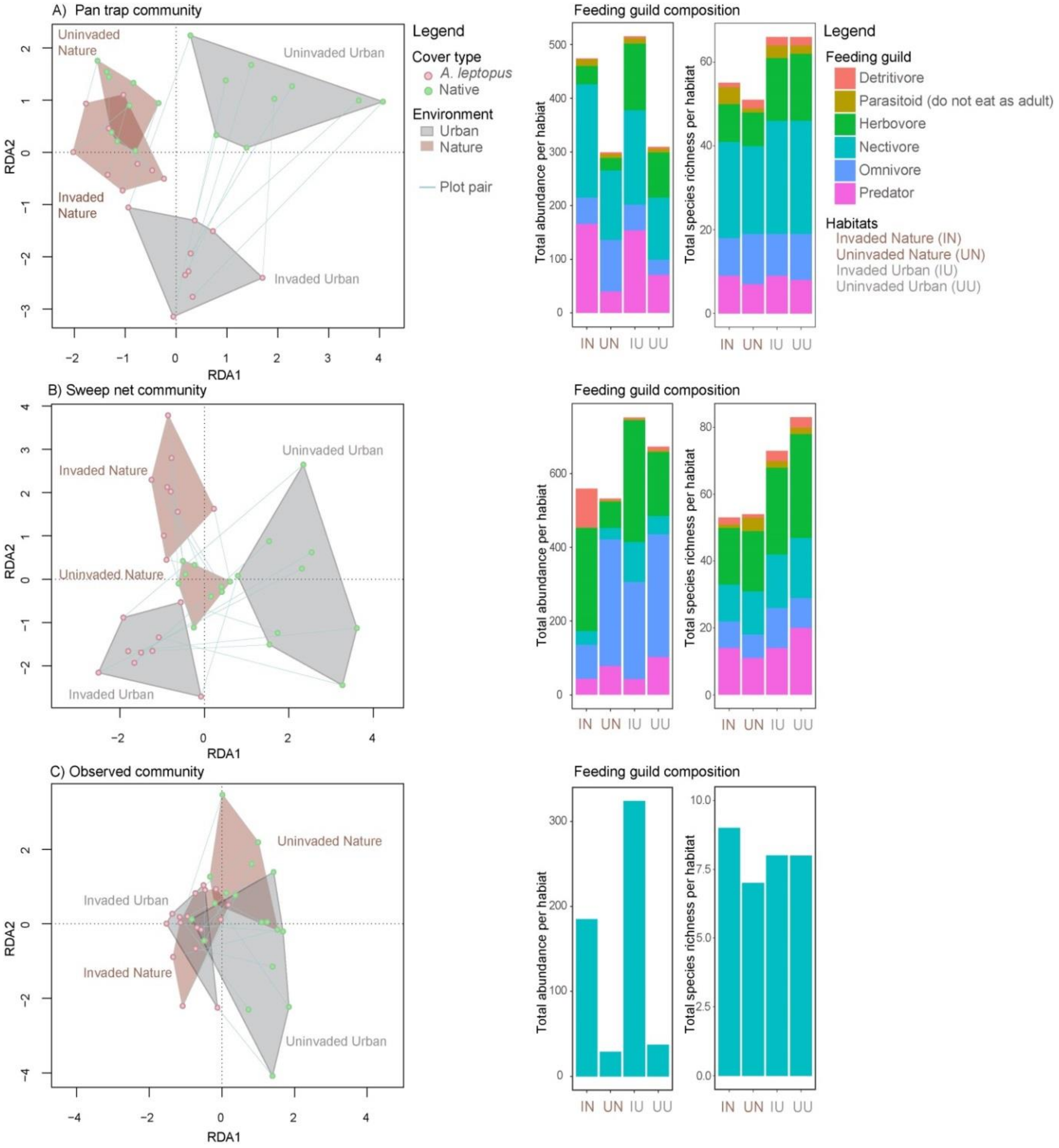

Sampling methods	Development	Invasion	Development*Invasion
Morpho-species (all methods combined)	**$F_{1,35}$ = 1.4, *P* < 0.01**	**$F_{1,35}$ = 1.5, *P* < 0.001**	$F_{1,35}$ = 1.2, *P* = 0.08
Pan traps	**$F_{1,35}$ = 1.5, *P* < 0.001**	$F_{1,35}$ = 1.2, *P* = 0.08	$F_{1,35}$ = 1.1, *P* = 0.19
Sweep net	$F_{1,35}$ = 1.1, *P* = 0.15	**$F_{1,35}$ = 1.5, *P* < 0.001**	**$F_{1,35}$ = 1.2, *P* = 0.04**
Observational survey	$F_{1,35}$ = 0.9, *P* = 0.56	**$F_{1,35}$ = 2.9, *P* < 0.001**	$F_{1,35}$ = 0.8, *P* = 0.66

Figure S8. Redundancy analyses on taxonomic morpho-species composition per sampling method. We separated the samples obtained through pan trap sampling (A), sweep net sampling (B) and observational sampling (C) and ran partial redundancy analyses on the 3 community subsets with invasion, development and their interaction as fixed factors, while accounting for variation associated with weather circumstances (i.e., rainfall, cloud cover, wind speed) and flower density. The table presents the model output from the morpho-species level analysis that is featured in the main text of the article in red. Analyses per sampling method are shown below and statistically significant ($\alpha = 0.05$) results are **bold**.

Compositional differences between invaded and uninvaded plots are consistent for all sampling methods, though this difference is slightly less pronounced in pan trap samples. Development significantly affected the composition of pan trap and sweep net samples, the latter through a significant interaction. Compositional differences can be explained by the feeding guild compositions of the samples per invasion-development habitat category, with sweep net and observational methods having relatively high abundances herbivores and nectarivores, respectively, which are significantly more associated to *A. leptopus* invaded plots (see results of feeding guild composition analyses in main text). We can thus conclude that all samples contribute to our overall observed patterns.

S9.

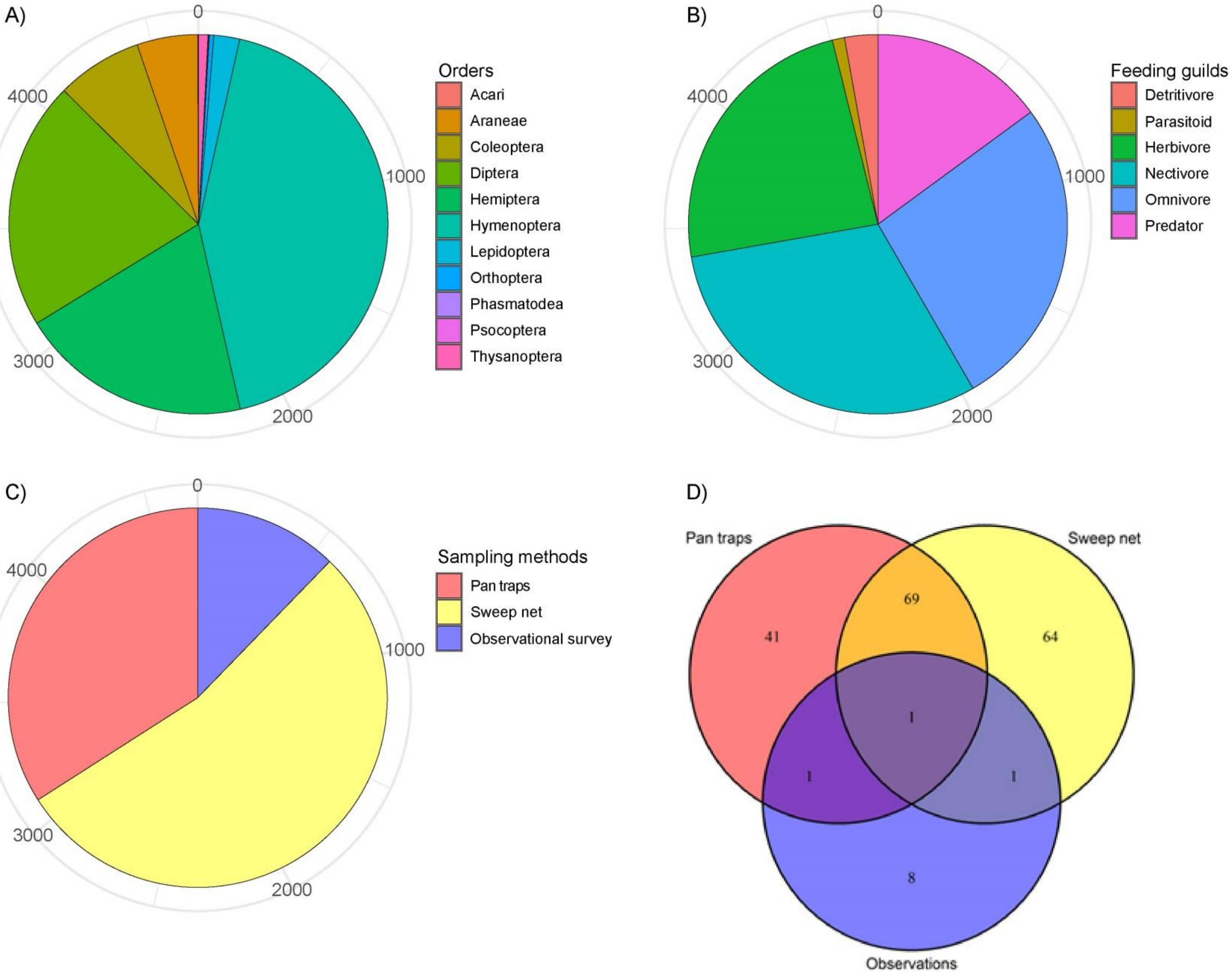

Figure S9. Composition of sampled arthropods. A total of 4690 arthropods were sampled, mostly including Hymenopterans, Dipterans, and Hemipterans (A). This indicates that our sampling techniques mostly targeted diurnal flying arthropods, rather than ground-dwelling arthropods. Furthermore, we sampled a varied, multi-trophic community, primarily including nectarivores, omnivores, herbivores and predators (B). Most arthropods were caught with a sweep net, followed by pan trap and observational sampling (C). These sampling techniques exhibited some species overlap (B), but complemented each other because they yielded high numbers of non-shared species that were exclusively sampled by a single technique.

S10

We included exponential Shannon diversity as dependent variable in the analyses featured in this manuscript, which indicated that the abundances of additional species in developed areas are distributed quite evenly over the species, significantly increasing the complexity of urban communities. However, in the review process of this manuscript, we also received the request of assessing the species-abundance distributions and evenness of species communities into more detail. To this end, we calculated community evenness and GAMBIN alpha values for the full species communities in our dataset (n=36), and tested them against effects of *A. leptopus* invasion, urban development, and their interaction, while accounting for plot pair in a LMM analysis. If disturbed (i.e., invaded and/or urban-developed) environments were characterized by uneven species distributions, consistent with the invasion of a (few) hyperdominant exotic arthropod species, this would result in severely unevenly distributed abundances over the existing species (i.e., low evenness and high GAMBIN alpha values (e.g., Fattorini et al., 2016). The mean evenness and alpha values and 95% confidence intervals are reported in the table below.

We find no indication of hyperdominance biasing our results. Even though *A. leptopus* invasion positively shifts alpha-values away from communities including many rare species to communities including many intermediately abundant species (0.87±0.29, z=3.009; P = 0.008), alpha values are relatively low and indicative of a log-series rather than a log-normal distribution (Table S10; Matthews et al., 2014). In comparison, communities that are dominated by a few species have α values around 8.0 (c.f., Fattorini et al., 2016) while the mean alpha values in Invaded Natural and Invaded Urban sites are 1.81 and 1.10, respectively (Table 1). In addition, evenness levels are consistent between invaded, uninvaded, developed and natural sites (all P-values > 0.30), and evenness values in all habitats are very high in general (Table S10; 0= total dominance; 1= total evenness), which would be highly unlikely if hyperdominance of a few species occurred in these sites.

Table S10. Species distributional indices per community.

Habitat type	Evenness		GAMBIN Alpha	
	**Mean**	95% CI	**Mean**	95% CI
Uninvaded Nature	**0.75**	0.61-0.89	**0.95**	0.47-1.43
Invaded Nature	**0.75**	0.66-0.85	**1.81**	1.15-2.48
Uninvaded Developed	**0.80**	0.74-0.86	**0.87**	0.51-1.24
Invaded Developed	**0.72**	0.65-0.79	**1.10**	0.78-1.43

References:

Matthews, T. J., Borregaard, M. K., Ugland, K. I., Borges, P. A., Rigal, F., Cardoso, P., & Whittaker, R. J. (2014). The gambin model provides a superior fit to species abundance distributions with a single free parameter: evidence, implementation and interpretation. *Ecography*, *37*(10), 1002-1011. https://doi.org/10.1111/ecog.00861

Fattorini, S., Rigal, F., Cardoso, P., & Borges, P. A. (2016). Using species abundance distribution models and diversity indices for biogeographical analyses. *Acta oecologica*, *70*, 21-28. https://doi.org/10.1016/j.actao.2015.11.003

S11.

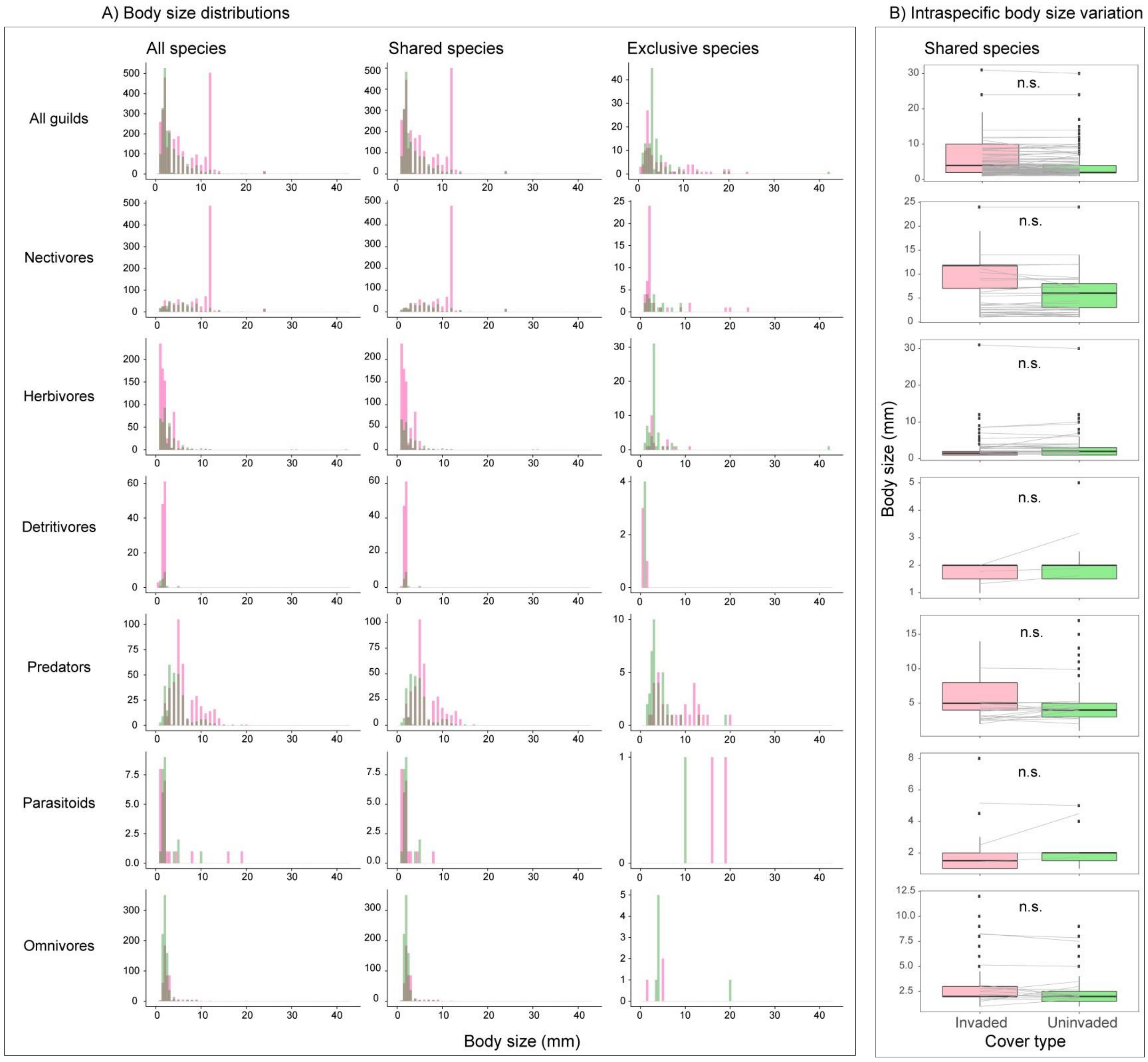

Figure S11. Differences in arthropod body size between *A. leptopus*-invaded (pink) and uninvaded (green) plots for all arthropods (top row) and per feeding guild (rows 2-7). A) Histograms of body size distributions. Figures in the first column show all species within the focal sample, in the second column show shared species that inhabit invaded as well as uninvaded plots, and figures in the third column illustrate species that occur exclusively in invaded (pink) uninvaded (green) habitat types.

- **All species**: The CWM body size increase in *A. leptopus*-invaded sites across all species, as featured in main text of this paper, seems mostly due to an increase in abundance of large 'shared' species between invaded and uninvaded plots. Some large species exclusively occur in invaded sites though in very low abundances. Therefore, CWM body size values could not have been overly affected by these *A. leptopus*-specific species.
- **Individual feeding guilds**: Within feeding guilds, nectarivores, predators, omnivores and parasitoids show a positive shift in overall body size distributions after *A. leptopus* invasion. These positive changes seem mostly due to the high abundance of large shared species, but *A. leptopus* also attracted low abundances of new, relatively large predatory, nectarivorous, and parasitoid species to the plots.

B) Intraspecific size differences among 'shared' species that inhabit both invaded and uninvaded sites. Only arthropods within sweep net and pan trap samples were included in these figures and associated statistical tests (n=4115), because we physically measured the body sizes of these individuals. Significance scores resulted from running linear mixed effects models for all arthropods and per feeding guild with log-transformed individual body size as dependent variable, invasion, development and their interaction as independent variables, and random factors of morpho-species, sampling method, and plot nested in plot pair: *log(body size) ~ Development * Invasion + (1|morphospecies) + (1|sampling method) + (1| Pair/Plot).*

Neither *A. leptopus* invasion, nor urban development significantly affected within-species body size in any model. Within the figure, the mean sizes of every species in both habitats is connected with a grey line, illustrating that there is no consistent increase in body size in invaded plots that would account for the community-weighted mean body size increase that we detected in our analyses (main text of this paper). Thus, *A. leptopus* does not seem to provide a better quality habitat for these species enabling them to grow bigger.
